# Vulnerability to Financial Scams Among Older Adults: Cognitive and Psychosocial Factors

**DOI:** 10.1093/geroni/igaa057.1447

**Published:** 2020-12-16

**Authors:** Scott Beach, Sara Czaja, Richard Schulz, David Loewenstein, Peter Lichtenberg

**Affiliations:** 1 University of Pittsburgh, Pittsburgh, Pennsylvania, United States; 2 Weill Cornell Medicine, New York, New York, United States; 3 University of Miami, Miami, Florida, United States; 4 Wayne State University, Detroit, Michigan, United States

## Abstract

This paper presents baseline results from a longitudinal study funded by National Institute on Aging examining financial exploitation (FE) among 720 White, African-American, and Hispanic adults age 60 and older. Our proposed conceptual model links socio-demographics, cognitive function, financial skills, and psychosocial factors to both scam exposure and vulnerability, which increases risk for experienced FE. This paper focuses on correlates of scam vulnerability, using a novel measure in which participants read six randomly ordered “scam” scenarios (investment opportunity; Medicare phishing and prescription drug fraud; health product; sweepstakes; telemarketing) and provide credibility ratings for each (1 = Not at all credible; 10 = Extremely credible). Ratings are coded “0” (credibility rating 1); “1” (ratings 2-4); “2” (ratings 5-10) and summed, with total “vulnerability” scores ranging from 0-12. Preliminary results from ~500 participants (Mean age = 73.6) show support for the proposed model. Older adults with poorer cognitive function in multiple domains (memory; reasoning ability; processing speed; other aspects of executive function; and crystallized intelligence); lower financial literacy; lower numeracy; those reporting difficulty with day-to-day financial management; and those performing worse on web-based banking skills tests were more vulnerable to scams. Scam vulnerability was a also higher among those with lower social integration; higher social isolation and loneliness; higher impulsivity; and higher ratings of depression. Use of “scam scenario” credibility ratings shows promise as a simple assessment approach for FE vulnerability. Understanding multiple pathways to FE is important to advance theory and for development of interventions to minimize risk among older adults.

